# Trust, but Verify: Comment on "‘Part of the Solution’: Food Corporation Strategies for Regulatory Capture and Legitimacy"

**DOI:** 10.34172/ijhpm.2022.7008

**Published:** 2022-02-26

**Authors:** Luke N. Allen

**Affiliations:** London School of Hygiene and Tropical Medicine, London, UK.

**Keywords:** Commercial Determinants of Health, Health Policy, Food Industry, Big Food

## Abstract

According to Lacy-Nichols and Williams, the food industry is increasingly forestalling regulation with incremental concessions and co-option of policy-making discourses and processes; bolstering their legitimacy via partnerships with credible stakeholders; and disarming critics by amending their product portfolios whilst maintaining high sales volumes and profits. Their assessment raises a number of fundamental philosophical questions that we must address in order to form an appropriate public health response: is it appropriate to treat every act of corporate citizenship with cynicism? If voluntary action leads to better health outcomes, does it matter whether profits are preserved? How should we balance any short-term benefits from industry-led reforms against the longer-term risk stemming from corporate capture of policy-making networks? I argue for a nuanced approach, focused on carefully defined health outcomes; allowing corporations the benefit of the doubt, but implementing robust binding measures the moment voluntary actions fail to meet independently set objectives.

## Frenemies

Lacy-Nichols and Williams argue that food corporations have learned to supplement tried-and-tested oppositional approaches to public health regulation with new conciliatory tactics.^1^ This comment seeks to spark lively debate by posing challenging questions about the generalisability of the authors’ findings, the moral criteria against which industry actions should be judged, and the practical implications for future engagement with industry.

Lacy-Nichols and Williams’s central thesis is that the turn of the millennium represented an inflection point in corporate strategy, with overt and vigorous opposition transitioning to appeasement and co-option under the banner of ‘constructive partnership.’ The authors offer detailed and comprehensive case study evidence for the shifting tone of industry rhetoric and present plentiful examples of self-regulation initiatives, public-private partnerships, and reformulation pledges from major companies over the past two decades, starting with seminal *mea culpa* moments from Kraft and Coke.^2,3^

What turned the tide? Encouragingly for public health academics, the proliferation of well-conducted research on the negative health impact of ultra-processed, sugar sweetened, high-salt content and trans-fat laden foods established an evidence base that was too convincing to ignore. Sowing doubt helped to stave off regulation for a while but arguing that trans fats & co were harmless eventually became untenable. As the burgeoning evidence base broke into public consciousness and health campaigns exposed nefarious industry tactics,^4,5^ the strategy of continued vociferous opposition attracted mounting risks: to reputation, credibility, and sales. Policy-maker sabre-rattling spooked investors and companies were forced to respond – publicly demonstrating that they understood the potential harms and would strive to mitigate them.^1^

## A New Approach: Killing With Kindness

 Lacy-Nichols and Williams argue that the food industry has worked hard to manage this process in a way that maximises their influence over policy-making processes, with efforts falling into three main components. The first is using self-regulation to forestall, co-opt, and neuter hostile regulatory environments. The authors see ‘ratcheting up’ of industry-led reforms, concessions and escalating accommodations as efforts to “acquire and secure corporate rule-making power.”

The second component is proactively building relationships with credible stakeholders in order to bolster credibility. Collaborations can earn praise from erstwhile critics and contribute to brand ‘health-washing.’ It is important to note that many companies continue to oppose public health regulations, but they have learned to farm out oppositional stances to distant front groups whilst they retain a credible public image and voice conciliatory overtures.^6^ Engaging with a widening network of partners is also about hedging: it is a good idea to have ‘multiple political access points’ if there is a future risk of exclusion from policy-making processes.

 The third component is changing product portfolios to disarm criticisms, through reformulation and portfolio diversification. An example would be Coca-Cola acquiring Innocent Smoothies and developing Coke Zero. Often companies do not reformulate their original offending product (eg, classic Coke). They may also only reformulate a limited number of products, not apply reforms to products sold in jurisdictions with softer regulatory environments, or substitute one harmful macronutrient for another.

## Fifty Shades of Grey: Appreciating Complexity and Heterogeneity

Industry-watchers will recognise all of these tactics, and there is ample evidence that food companies – as well as tobacco, alcohol and gambling companies – have increasingly repositioned themselves as responsible partners working alongside communities and governments to tackle problems they help to cause. Lacy-Nichols and Williams assiduously justify their assertions with over 140 references covering two decades of high-quality commercial determinants research. However, whilst making an important contribution to the literature, their paper raises important moral questions without surfacing the authors’ underlying philosophical paradigm. Their paper also treats all food companies as a homogenous and coordinated bloc, lumping smaller businesses together with transnational giants. This engenders unhelpful conspiratorial overtones that belie the messy and fragmented reality of corporate strategy development. Granted, the food sector is a consolidated oligopoly and shorthand references to ‘industry tactics’ are helpful, however as the field of commercial determinants matures, we need to adopt more nuanced analytic frameworks that recognise the heterogeneous reality.^7^

The major players in the food industry employ thousands of people, working in hundreds of teams across multiple regions and jurisdictions. Their health impact is manifest through multiple different channels, depending on their business model, ethos, employment practices, supply chains etc. Executives sometimes commit to acts that reduce earning potential because they align with their or the company’s non-pecuniary values. Robust commercial determinants scholarship should reflect the fact that every company is unique and exerts a unique health impact. Furthermore, whilst the ‘guilty until proven innocent’ approach is wholly appropriate for serial offenders, it stands at odds with moral norms^8^; calls out the worst in firms; and leads us away from a risk-proportionate approach.

## So What? and What Matters?

 If a company diversifies its portfolio, reformulates unhealthy products, and stops marketing to children so that consumption of its unhealthiest products declines, does it matter that their motives are self-serving? Should we judge companies based on intentions, actions, or outcomes? If diet quality improves as a result of industry self-regulation, does it matter if profits rise? What criteria should we use to judge the actions of individual companies?

Lacy-Nichols and Williams implicitly espouse both Aristotelian virtue ethics^9^ and deontological stances^10^ when a consequentialist paradigm^11^ is likely to offer the most purchase.^12,13^ For the public health community I would argue that *health outcomes* are what matter ie, the level and distribution of dietary intakes matters more than the means of achievement or underlying motivations.

## Doveryay, No Proveryay

 This consequentialist reckoning must reconcile short- and long-term time horizons, acknowledging that whilst industry concessions can improve short-term health outcomes these can come at the expense of growing influence over policy-making processes which threaten health outcomes in the medium and longer term. Vitally, voluntary actions that achieve health gains are only valuable if they do not undermine, forestall, or dilute more effective forms of regulation.

 If we move to judge industry actions according to outcomes, then profits and corporate image exchange any intrinsic moral value for purely instrumental value – ie, they would only concern us if/when they harm health.

How should we – the public health community – respond to ‘good citizenship’ initiatives? This question touches on even more fundamental Hobbesian themes^14,15^ about human nature. Personally, I refuse to believe that every single initiative from every single firm is launched from purely Friedmanian motives (“the social responsibility of business is to increase its profits”^16^) and although we have won the right to react with cynicism to the major transnationals, I believe our societies should use the least restrictive public health measures to achieve a given end^17^ and make room for all companies to ‘do the right thing’ – albeit with iron-clad regulatory backups that are triggered when pre-set targets are not met.

 We should judge proposed commercial activities according to their holistic health impacts, modelled using complexity-informed political economy frameworks that can balance reputational and hegemonic gains against putative health benefits in the broadest sense eg, including environmental impact, work conditions, tax implications etc.

Key to this approach is the establishment of clear, evidence-based, specific, measurable, ambitious, time-bound targets, backed by a willingness to introduce binding legislation the very moment it becomes clear that industry-led action will not meet these targets. If companies want to take the lead with voluntary reform this should be welcomed insofar as the actions demonstrably meet these *a priori* goals set by national policy-makers and assessed by fully independent evaluators. This approach can be summarised by the Russian proverb *doveryay, no proveryay* – ‘trust but verify’ – popularised during the Soviet-American nuclear disarmament talks.

Two major limitations of judging corporate actions on a case-by-case basis are the resources involved (time, expertise, independent personnel, money) and a challenge shared with system dynamic modelling of complex adaptive systems – where to draw the boundaries? Including environmental, tax and labour elements in corporate impact assessments sounds great, and these elements definitely influence health, but holistic assessments take an incredibly long time to do well and often rest on multitudinous assumptions. Then there is also an issue in defining the appropriate commercial unit of analysis; should we be assessing the impact of Coke’s national office, a single product, or the entire transnational corporation? Baum and colleagues have produced a helpful multi-dimensional framework to guide the development of such impact assessments^18,19^ however an important question remains; why should the burden of proof fall on public health researchers? And who should pay for all this work? Responsibility for health protection falls to the Health Ministry, but there is probably a role for extended mandatory disclosures of industry data to health ministries in order to reduce the burden of assessment.

## An Awkward Embrace

A final issue is whether and how to engage corporate actors in policy-making processes – especially those around setting national targets that will be used to judge industry actions and in designing the regulatory ultimatum (what legislation and when it should be triggered?). Clearly there is a huge conflict of interest allowing companies to set or influence rules, however policy-makers are unavoidably dependent on industry data to conduct accurate situational analyses and model the impact of differing targets, thresholds and policy scenarios. Policy-makers themselves may also have conflicts of interest that influence their decisions. Even when they don’t, we need to recognise that companies wield enormous power.^20-25^ High levels of corporate permeation is associated with lower levels of health policy implementation,^26^ and emerging evidence suggests that countries with weaker regulatory protections against overt and covert corporate financial donations to policy-makers are systematically under-implementing policies to restrict the sale and advertising of tobacco, alcohol and junk food.^27^

 The safest option is to take the tobacco approach – completely insulating all policy discussions from industry representatives. At the other end of the spectrum is full engagement, proactively welcoming those businesses that make the right noises to take a seat at the table. I agree with Lacy-Nichols and Williams that open partnership fosters social rehabilitation, legitimisation and opportunities for co-option that pose major strategic risks in the long term and we need to be very cautious about this approach.

A degree of cooperation is probably unavoidable (and desirable) for carefully circumscribed issues, through controlled and independently monitored mechanisms, to meet pre-specified targets over which industry has no input. I suggest that companies should never be involved in setting the targets that they will be held to, or in deciding what regulatory action will be enforced should the targets be missed. It is possible to be strict about these red lines whilst engaging industry on other issues without either vilifying or valorising. Revolving door appointments and legal lobbying avenues make it impossible to completely eliminate conflicts of interest, however sensible principles can mitigate industry influence. These include establishing clear *a priori* policy goals for each issue at hand, conducting thorough due diligence before agreeing to any engagement, considering the use for formal legal arrangements, and situating engagement activities within established statutory and regulatory frameworks designed to protect the public and the health agency’s reputation.^28^ As previously mentioned, mandatory reporting of sales and nutritional data would also help monitoring and enforcement.

 What might this look like in practice? A government would set a dietary goal such as salt consumption <5 g/day/person, or a maximum of 5 g sugar/100 mL in beverages. Next, it would set an ambitious reformulation timetable for industry. Industry would be invited to provide data, but have no say over the timetable. Then mandatory regulation would be set up (eg, banning the sale of transgressing products or imposing fines on producers) but only triggered if/when industry failed to voluntarily meet the government’s pre-set milestones. Regular monitoring would be conducted by an independent body.

Examples of *what not to do* abound. Canada set voluntary trans fat reduction targets in 2007 with the promise to regulate if pre-specified targets were not met within two years. The government failed to insulate the monitoring process from industry and still has not delivered on its ultimatum.^29^ Portugal’s ‘Food Industry Co-Agreement’ set ambitious sugar, salt, and trans fats reformulation targets in 2019, however industry negotiators managed to dilute the targets and extend the deadlines.^30^ The United Kingdom had an unusually good experience with voluntary salt reduction,^31^ but failed to replicate this with sugar.^32^ These examples highlight the power of industry and remind us that the safer option is to go straight to regulation where there are any concerns about the government’s ability to insulate processes from industry influence.

In summary, the least restrictive measure should be used to attain a public health goal. However, voluntary actions must always work towards prespecified targets that are independently set and monitored. *Ineffective* voluntary action should never be allowed to forestall mandatory action. Given that voluntary approaches tend to be less effective than statutory approaches^33,34^ we should always have legislation ‘waiting in the wings’ – ready to be triggered the moment *a priori* targets are missed.

## Conclusion: Hope Beyond Blanket Cynicism

 Lacy-Nichols and Williams have deftly delineated the “broad contours of the food corporate strategic culture of accommodation” over the past 20 years. Whilst we have troves of data on what companies do and say, we have much dimmer insight into the calculus and motivations that underlie these actions.

 It is fairly safe to assume that for-profit enterprises pursue actions that are expected to deliver a financial return; directly or indirectly through enhanced corporate image and/or increased power. We should expect companies to deploy the minimum viable voluntary concessions, at the last possible moment that will forestall more effective legislative action.

However, we should recognise that not all companies are alike, and shareholders and chief executives are increasingly willing to sacrifice (a highly variable degree of) profit in order to achieve other non-financial goals.^35^ In other words; not all companies are all-bad all of the time. Our current ‘guilty until proven innocent’ approach does not align with core public health principles and I would welcome a change in tone that calls on companies to deliver on their pledges and gives them carefully prescribed space to do so. I hope this reflects eyes-open appreciation of nuance rather than naïveté. Granted, given that major ultra-processed food companies have been given ample space to ‘do the right thing’ but delivered meagre results, blanket cynicism is wholly warranted. But I wonder if we can do better?

 I have also argued that motives do not matter as long as we take a long and holistic view of health outcomes. I willingly concede that it can be very difficult to estimate the long-run impacts of increased profits, growing market share, and reputational enhancement, so that co-option and legitimisation are major medium and long-term threats. Scenario modelling should be hawkish, defaulting to worst-case models for distant outcomes.

 Our research community plays an essential role in developing the frameworks, mechanisms and tools that could allow companies latitude to implement meaningful voluntary reforms in the context of bona fide partnerships, backed by no-nonsense regulatory safeguards. This approach would put governments firmly in charge of the regulatory agenda and hold corporate actors to ambitious and externally set targets, whilst welcoming them to make good on their claims as responsible global citizens. It may be that this nuanced and risk-proportionate approach is not worth the potential costs, and I invite discussion of where it would and would not be appropriate as our field continues to grow and develop.

## Ethical issues

 Not applicable.

## Competing interests

 Author declares that he has no competing interests.

## Author’s contribution

 LNA is the single author of the paper.
